# Systematic review and meta-analysis of published randomized controlled trials comparing purse-string *vs* conventional linear closure of the wound following ileostomy (stoma) closure

**DOI:** 10.1093/gastro/gou038

**Published:** 2014-07-10

**Authors:** Muhammad Shafique Sajid, Muhammad I. Bhatti, William FA. Miles

**Affiliations:** ^1^Department of General, Endoscopic & Laparoscopic Colorectal Surgery, Western Sussex Hospitals NHS Foundation Trust, Worthing Hospital, UK and ^2^Department of General & Colorectal Surgery, Queen Elizabeth Hospital King’s Lynn NHS Foundation Trust, UK

**Keywords:** stoma closure, ileostomy closure, purse-string wound closure, linear wound closure

## Abstract

**Objective:** The objective of this article is to systematically analyse the randomized, controlled trials comparing the effectiveness of purse-string closure (PSC) of an ileostomy wound with conventional linear closure (CLC).

**Methods:** Randomized, controlled trials comparing the effectiveness of purse-string closure *vs* conventional linear closure (CLC) of ileostomy wound in patients undergoing ileostomy closure were analysed using RevMan®, and the combined outcomes were expressed as risk ratio (RR) and standardized mean difference (SMD).

**Results:** Three randomized, controlled trials, recruiting 206 patients, were retrieved from medical electronic databases. There were 105 patients in the PSC group and 101 patients in the CLC group. There was no heterogeneity among included trials. Duration of operation (SMD: −0.18; 95% CI: −0.45, 0.09; z = 1.28; *P <* 0.20) and length of hospital stay (SMD: 0.01; 95% CI: −0.26, 0.28; z = 0.07; *P <* 0.95) was statistically similar following both approaches of ileostomy wound closure. The risk of surgical site infection (OR, 0.10; 95% CI: 0.03, 0.33; z = 3.78; *P <* 0.0001) was significantly reduced when ileostomy wound was closed using PSC technique.

**Conclusion:** PSC technique for ileostomy wound is associated with a reduced risk of surgical site infection apparently without influencing the duration of operation and length of hospital stay.

## INTRODUCTION

The loop ileostomy is a commonly used defunctioning stoma that is often inserted to minimise the consequences of anastomotic leak following low and ultra-low anterior resection, ileo-anal anastomosis, ileal pouch-anal anastomosis, and in circumstances where reversible patient factors increase the risk of an anastomotic dehiscence [1–6]. However, closure of ileostomy is associated with significant operative morbidity, varying from 18–67% [7, 8], and mortality is reported up to 4% [9]. Surgical site infection (SSI) is the most common complication, ranging up to 41% [7–10]. Various ileostomy wound closure techniques have been employed to reduce this appallingly higher level of SSI in these patients, such as primary continuous or interrupted stitch wound closure, primary closure with drain, loop primary closure, delayed-primary closure, secondary closure and purse-string closure (PSC) [11]. The most highly rated technique for reducing the incidence of SSI following ileostomy closure is PSC. The objective of this article is to systematically analyse the randomized, controlled trials comparing the effectiveness of PSC *vs* conventional linear closure (CLC) of ileostomy wound in patients undergoing ileostomy closure.

## METHODS

A search was conducted of standard electronic databases—such as MEDLINE, EMBASE, and the Cochrane Library—for randomized, controlled trials comparing the effectiveness of PSC *vs* CLC of ileostomy wound. The MeSH terms published in the Medline library were used to ‘hit’ upon the relevant trials. No restrictions for language, gender, sample size or place of study origin were entered for the search. Boolean operators (AND, OR, NOT) were appropriately utilized to narrow and widen the search results. The published titles from the resultant search were scrutinized closely and their suitability for potential inclusion into this study was assessed. The references from selected published articles were also checked as a further search tool, to find additional studies. For inclusion in the meta-analysis, a study had to meet the following criteria: (i) randomized, controlled trial; (ii) comparison between PSC and CLC; (iii) evaluation of surgical site infection; (iv) main outcome measures reported preferably as an intention-to-treat (ITT) analysis; and (v) trials in surgical patients who had undergone ileostomy or jointly colostomy closure for any indication. Two reviewers, using a pre-defined meta-analysis form, extracted data from each study, which resulted in high and satisfactory inter-observer agreement. The extracted data contained information regarding the name of the authors, title of the study, journal in which the study was published, country and year of the study, treatment regimen, length of the therapy, testing sample size (with sex differentiation if applicable), the number of patients receiving each regimen and, within the group, the number of patients who succeeded and the number of patients who failed the allocated treatment, the patient compliance rate in each group, the number of patients reporting complications and the number of patients with absence of complications in each arm.

The RevMan 5.2 software package [12, 13], provided by the Cochrane Collaboration, was used for the statistical analysis to achieve a combined outcome. The risk ratio (RR) with a 95% confidence interval (CI) was calculated for binary data, and the standardized mean difference (SMD) with a 95% CI was calculated for continuous variables. The random-effects model [14, 15] was used to calculate the combined outcomes of both binary and continuous variables. Heterogeneity was explored using the chi-squared (chi^2^) test—with significance set at *P <* 0.05—and was quantified using the I^2^ test, with a maximum value of 30% identifying low heterogeneity [16]. The Mantel-Haenszel method was used for the calculation of RR under the random effect models [17]. In a sensitivity analysis, 0.5 was added to each cell frequency, for trials in which no event occurred in either the treatment or control group, according to the method recommended by Deeks *et al.* [18]. If the standard deviation was not available, then it was calculated according to the guidelines of the Cochrane Collaboration [12]. This process involved assumptions that both groups had the same variance—which may not have been true—and variance was either estimated from the range or from the *P*-value. The estimate of the difference between the two techniques was pooled, depending upon the effect weights in results determined by each trial estimate variance. A forest plot was used for the graphical display of the results. The square around the estimate stood for the accuracy of the estimation (sample size), and the horizontal line represented the 95% CI. The methodological quality of the included trials was initially assessed using the published guidelines of Jadad *et al.* and Chalmers *et al.* [19–20]. Based on the quality of the included randomised, controlled trials, the strength and summary of the evidence was further evaluated by GradePro® [21], a tool provided by the Cochrane Collaboration. Surgical site infection was decided upon as the primary endpoint in this study. Secondary endpoints included duration of operation and length of hospital stay.

## RESULTS

**Figure 1:**
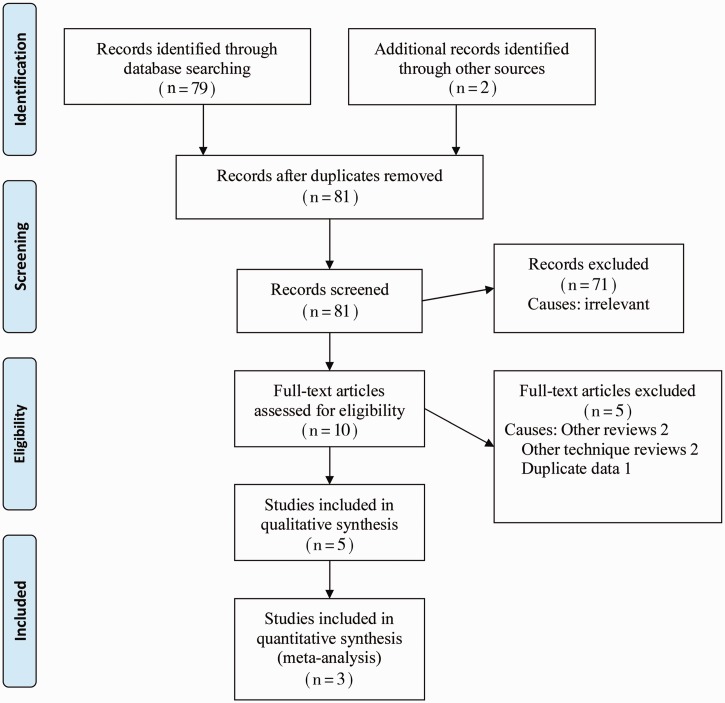
PRISMA flow diagram.

The PRISMA flow chart to explain the literature search strategy and trial selection is given in Figure 1. Three randomized, controlled trials [22–24], encompassing 206 patients, were retrieved from the search of medical electronic databases. There were 105 patients in the PSC group and 101 patients in the CLC group.

### Methodological quality of included studies

**Figure 2:**
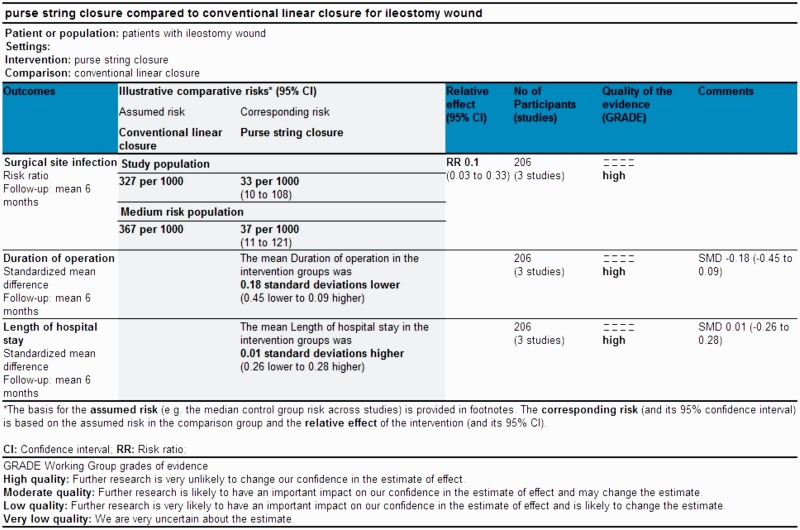
Strength and summary of the evidence analysed on GradePro®.

According to Jadad *et al.* and Chalmers *et al.* [19, 20] the quality of included trials was good, due to the satisfactory utilization of randomization techniques. In addition, there was adequate reporting of power calculation, allocation concealment and intention-to-treat analysis. However, blinding was absent in the majority of trials. Based on the quality of included randomized, controlled trials, the strength and summary of evidence analysed on GradePro® is given in Figure 2.

### Surgical site infection

**Figure 3:**
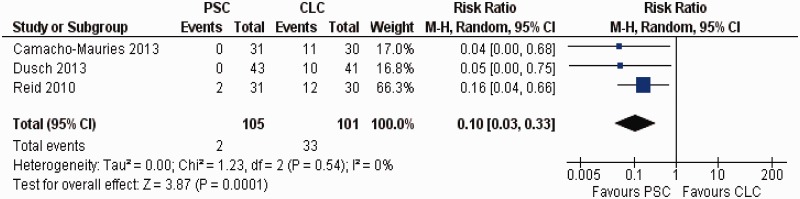
Forest plot for surgical site infection following purse-string closure (PSC) *vs* conventional linear closure (CLC) in patients undergoing ileostomy closure.

There was no heterogeneity (Tau^2 ^= 0.00; chi^2 ^= 1.23; df = 2, [*P =* 0.54]; I^2 ^= 0%) among trials. In the random effects model (RR: 0.1; 95% CI: 0.03–0.33; z = 3.87; *P =* 0.0001) (Figure 3), the PSC technique of ileostomy wound closure was associated with a reduced incidence of SSI compared with CLC technique.

### Duration of operation

**Figure 4:**
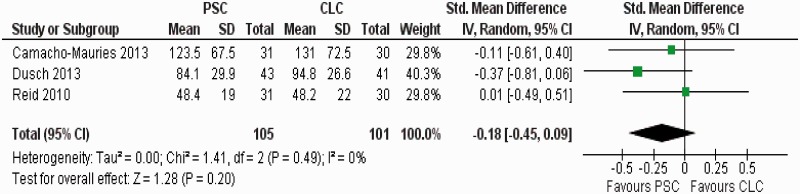
Forest plot for duration of operation following purse-string closure (PSC) *vs* conventional linear closure (CLC) in patients undergoing ileostomy closure.

There was no heterogeneity (Tau^2 ^= 0.0; chi^2 ^= 1.41; df = 2, [*P =* 0.49]; I^2 ^= 0%) among trials. In the random effects model (SMD: −0.18; 95% CI: −0.45–0.09; z = 1.28; *P =* 0.20) (Figure 4), the duration of operation was also shorter in the PSC group; however, the statistical significance was not reached.

### Length of hospital stay

**Figure 5:**
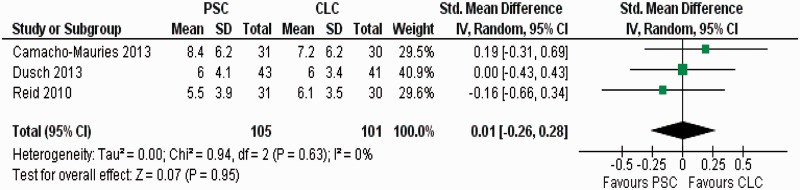
Forest plot for length of hospital stay following purse-string closure (PSC) *vs* conventional linear closure (CLC) in patients undergoing ileostomy closure.

There was no heterogeneity (Tau^2 ^= 0.0; chi^2 ^= 0.94; df = 2, [*P =* 0.63]; I^2 ^= 0%) among trials. In the random effects model (SMD: 0.01; 95% CI: −0.26–0.28; z = 0.07; *P =* 0.95) (Figure 5), the length of hospital stay was similar in the PSC and CLC groups.

## DISCUSSION

This review demonstrates that the PSC technique for ileostomy wound is associated with a 90% reduced risk of SSI, apparently without influencing the duration of operation or length of hospital stay. The findings of this review are in concordance with previously published studies. A recently published systematic review by Li *et al.* on 15 studies showed that circular closure of stoma wounds was the best skin closure technique in terms of SSI rate, but the quality of supporting evidence is limited, precluding definite conclusions [11]. Although the authors openly acknowledged the limitation of their study, they failed to demonstrate the reason for exclusion of two high-quality and recently published, randomized, controlled trials in their meta-analysis [22, 23]. In addition, their study reported the combined analysis of randomized, non-randomized and case-controlled studies, which itself is a serious methodological flaw in performing and reporting a systematic review. Another review, published by McCartan *et al.*, reported the meta-analysis of six studies showing that the PSC of stoma wounds was associated with an 80% reduction in SSI, with no negative effect on length of hospital stay or long-term cosmetic outcome [25]. However, McCartan *et al.* reported the combined analysis of only two randomized, controlled trials and four case-controlled studies, limiting the credibility of reported conclusion. Our study is the combined analysis of three randomized, controlled trials of good quality, without contamination from any other non-randomized or case-controlled study, and therefore the reported evidence may be considered conclusive and unbiased.

Understandably, the purse-string approximation technique of ileostomy wound closure offers several advantages due to its nature. Purse-string closure leaves a central drainage pit approximately one centimetre in diameter, which allows continuous drainage of exudative and suppurative fluid in this grossly contaminated wound, resulting in a seamless process of granulation and healing of the wound. Furthermore, once the sub-cuticular stitch is either absorbed or removed and the central pit is filled with nicely granulating tissue, it is covered by surrounding epidermis, leading to a wound with better cosmetic appearance. In contrast, linear closure does not offer the drainage of suppurative fluid, resulting in higher risk of SSI, abscess formation, and under-granulating wound which, in that environment, leads to delayed healing with bigger scar that is cosmetically poor. Trials comparing open *vs* closed wound techniques of ileostomy wound closure may be an interesting idea but they have not yet been reported in the medical literature; however, a recently published meta-analysis has reported six varied techniques of ileostomy wound closure incorporating different open wound techniques, and closed wound techniques with and without drainage, but substantially confirmed the superiority of PSC technique.

Three included RCTs in this systematic review randomized, controlled trials [22–24] evaluated SSI as primary or secondary outcomes according to the pre-trial analysis strategy. The use of SSI as primary or secondary endpoints following PSC or CLC was well targeted because SSI is a major burden of morbidity in patients undergoing ileostomy closure. This outcome was thoroughly investigated and adequately reported in included RCTs in this systematic review randomized, controlled trials. The summated outcome of this variable was conclusive and may be considered adequate. Based on the technique of randomisation, allocation concealment, power calculations, single or double blinding and reporting of intention-to-treat analysis in majority of the included trials were considered methodologically adequate. In the combined outcome of these trials—in conjunction with the results of previously published studies [11, 25]—there seems to be sufficient evidence to support the conclusion that PSC is associated with a reduced incidence of SSI.

The present review also has some limitations. Studies included in this review that recruited a small number of patients, may not have had sufficient power to reveal small differences in outcomes. Due to smaller numbers of patients and fewer trials on this subject, it is still unwise to generally apply the results of this study to all groups of patients undergoing stoma closure. Future research should be aimed at evaluating the PSC of stoma wound in ileostomy and colostomy closure patients separately, to find which group might benefit more. The influence of adjunctive measures, such as liberal or restrictive use of antibiotics, presence of cancer and other co-morbid conditions at the time of stoma closure, should also be taken into account before recommending the routine use of PSC for stoma wound closure. A major, multicentre, high-powered randomized, controlled trial is desirable to validate the findings of this review and, until then, the present study may assist colorectal surgeons in making decisions on which technique should be adopted for stoma wound closure.

*Conflict of interest statement:* none declared.
